# Theoretical and Experimental Study of Phonon Spectra of Bulk and Nano-Sized MoS_2_ Layer Crystals

**DOI:** 10.1186/s11671-016-1808-8

**Published:** 2017-01-31

**Authors:** Anatoliy Mikhailovich Yaremko, Volodymyr Oleksandrovych Yukhymchuk, Yuriy Anatolijovych Romanyuk, Jan Baran, Marcel Placidi

**Affiliations:** 1 V.E Lashkaryov Institute of Semiconductor Physics NAS of Ukraine, Prospect Nauky 45, Kyiv, 03028 Ukraine; 2 Institute of Low Temperature and Structure Research of the PAS, ul. Okólna 2, Wrocław, 50-422 Poland; 3 Catalonia Institute for Energy Research (IREC), Jardins de les Dones de Negre 1, Sant Adrià de Besòs, Barcelona, 08930 Spain

**Keywords:** Layer crystals, Atomically thin crystals, Raman scattering, Phonons, Interlayer interaction, 74.25.Kc, 78.30.Fs, 78.66.Hf

## Abstract

Theoretical analysis of Raman scattering spectra (RS) for single-crystal MoS_2_ sample and atomically thin MoS_2_ sample consisting from one to few layers was performed in order to explain the change of MoS_2_ vibrations at transition from a monoatomic layer to a bulk crystal. Experiments have shown that changes of frequencies of the most intensive bands arising from the in-plane, ${E}_{2 g}^1$, and out-of-plane, *A*_1*g*_, vibrations, as a function of number *n* of layers looks differently. Thus, the frequency of *ω*(*A*_1*g*_) is increasing with growth of *n*, whereas the frequency of $\omega \left({E}_{2 g}^1\right)$ is decreasing. Such a change of the $\omega \left({E}_{2 g}^1\right)$ frequency was explained as the effect of “strong increase of the dielectric tensor when going from single layer to the bulk” sample. In the present work, we show that the reason of different dependences of frequencies can be related to both the van der Waals (vdW) interlayer interaction and the anharmonic interaction of noted fundamental vibrations with the corresponding combination tones (CT) of layer that manifests itself due to Fermi resonance in the layer. Overjumping of these phonon pairs (*s*, *s* ') owing to interlayer interaction, $\tilde{V}\left({}_{s, s\hbox{'}, q}^p\right)$, to other layers at growth of number *n*, results in the change of frequencies for each interacting pair of *A*_1*g*_ or ${E}_{2 g}^1$ symmetry. The alteration of pair frequencies depends on the ratio of constants $\tilde{V}\left({}_{s, s\hbox{'}, q}^p\right)$ describing the interaction of studied states *s* and *s* '.

**Electronic supplementary material:**

The online version of this article (doi:10.1186/s11671-016-1808-8) contains supplementary material, which is available to authorized users.

## Background

Investigations of electronic and vibration spectroscopic properties of layer type crystals have already been done quite a long time ago.

The new period of activity in the study of such crystals arose again when Novoselov et al [1] published their results related to graphene. The usage of their method allows to prepare a very thin crystal structure that has 1–10 atomic layers [1]. The layer type crystals MoS_2_ and MoSe_2_ are especially perspective in this sphere, as they show very interesting spectroscopy features, if the sample consists of only few atomic layers. In particular, electronic band structure of such crystals differs from the bulk ones, and they demonstrate very intensive luminescence [2, 3]. The number of works in which electronic and phonon properties of such type of crystal structures that are studied by spectroscopy methods significantly increased during the last years.

Recently, a detailed study of vibrational spectra of MoS_2_ layer crystals consisting of several layers, *n* = 1–6 (atomically thin crystals), and analysis of former results were made in [4, 5]. Strong signals of the in-plane $\left({E}_{2 g}^1\right)$ and out-of-plane (*A*_1*g*_) Raman modes were observed for all 1–6 layer samples. These modes exhibited well-defined thickness dependence, thus the frequencies of these two modes are shifting away from each other with increasing thickness. The behaviour of frequency shifts with the layer thickness as emphasized in [4] cannot be explained solely in the terms of a weak van der Waals (vdW) interlayer interaction.

The most striking is that frequency of the low frequency ${E}_{2 g}^1$ vibration decreases (red shifts), while that of the high frequency *A*_1*g*_ vibration increases (blue shifts) with the increasing of the sample thickness. Besides, the frequencies of both these modes converge to bulk values, when films have four or more layers.

Within a classical model for coupled harmonic oscillators [3], the frequencies of ${E}_{2 g}^1$ and *A*_1*g*_ modes are expected to increase as additional layers are added to form the bulk material from individual layers. That means, that the interlayer vdW interactions increase the effective restoring forces acting on the atoms. Therefore, the shift of *A*_1*g*_ mode observed in experiments of works [4, 5] with increasing of layer number seems to be in agreement with the theoretical prediction; however, the behaviour of the ${E}_{2 g}^1$ mode does not agree. The reason of such a behaviour of ${E}_{2 g}^1$ may be attributed [4] to the long-range Coulombic interlayer interaction [6, 7] what manifests as anomalous Davydov splitting (DS) [8, 9]. Similar features have also been observed in crystals of GaSe [6] and GaS [10]. Anomalous DS means that there are additional factors, besides of “pure DS effect”, which are displayed in experiment.

Indeed, not only vdW interlayer interaction, but also its combination with anharmonic interactions that occur in complex molecules or layers, can give rise to new features in dynamics of crystal lattice and observed in infrared (IR) and Raman spectroscopy (RS). Such approach was developed in a number of works [11–13] in 70-es of the last century, where not only DS effect but also its combination with intramolecular Fermi resonance (FR) was taken into account. Such effect is known as Fermi-Davydov (FD) resonance in molecular type crystals. It was shown in the cited works that frequencies of crystal vibrations and their intensities depend on treatment of the vdW inter-molecular interaction and intra-molecular anharmonicity. More complicated situation of displaying FD resonance with participation of lattice phonons was studied in crystals with hydrogen bond in which anharmonic constants can be greater than in the case of lattice phonons [14]. Therefore, we suppose that features observed in recently published Raman spectra [4, 5] can be explained in the framework of approach developed in following works [11–13]. However, the problem connected with the influence of weak interlayer interactions on strong *intralayer* interaction looks more complex in this case, because each layer is a two-dimensional crystal.

Therefore, in the present work, we at first consider the response of the bulk layer crystal on incident radiation, writing the Hamiltonian for the layer type crystal in a form which separates strong intralayer interactions and weak interlayer ones. Then, by variation of the vdW interaction value between layers (the effect of dielectric function [7] is considered to be included in both the intralayer interactions and in the vdW parameters), we can analyse the change of layer frequencies in the framework of the developed approach below.

## Methods

### Intensity of Raman Scattering

RS intensity is expressed by the imaginary part of Fourier component of Green function (GF) on tensor of susceptibility of crystal *χ*_*k*,*λ*,*k* ',*λ* '_ [15, 16].

1 $$ {\chi}_{k,\lambda . k\hbox{'},\lambda \hbox{'}}={\displaystyle \sum_{\alpha, \beta}{\overline{e}}_{\alpha}\left( k,\lambda \right){\overline{e}}_{\beta}^{\ast}\left( k\hbox{'},\lambda \hbox{'}\right)}{\chi}_{\alpha, \beta}\left( k\hbox{'}- k\right), $$

2 $$ {\chi}_{\alpha, \beta}\left(\overrightarrow{Q}=\overrightarrow{k}\hbox{'}-\overrightarrow{k}\right)={\displaystyle \sum_m{\chi}_{\alpha, \beta}\left(\overrightarrow{m}\right) \exp \left(- i\overrightarrow{Q}\overrightarrow{m}\right)} $$

Here, *ē*_*α*_(*k*, *λ*) is α-component of electric field *E*_*α*_(*m*) unit vector; *χ*_*α*,*β*_(*m*) are the components of susceptibility tensor of the *m*-th unit cell of a crystal.

For a layer type crystal, the tensor (2) has some specific features; in particular, two indexes must be used (*m* → *l*, *n*) to number the unit cells of a crystal: the first index (*l*) points out the number of layer and other one (*n*) numbers the unit cell in given layer. The wave vector is also convenient to present by two components oriented in layer, ${\overrightarrow{Q}}_n$, and normal to layer, ${\overrightarrow{Q}}_l$, respectively, so that $\overrightarrow{Q}={\overrightarrow{Q}}_l+{\overrightarrow{Q}}_n$. Then

3 $$ {\chi}_{\alpha, \beta}\left(\overrightarrow{Q}=\overrightarrow{k}\hbox{'}-\overrightarrow{k}\right)=\kern0.5em {\displaystyle \sum_{n, l}{\chi}_{\alpha, \beta}\left(\overrightarrow{n}+\overrightarrow{l}\right) \exp \left[- i\left(\overrightarrow{n}{\overrightarrow{Q}}_n+\overrightarrow{l}{\overrightarrow{Q}}_l\right)\right]} $$

Taking into account an identical crystal layers, making the Fourier transformation of Eq. (3) and inserting the results into Eq. (1), the expression for susceptibility of layer crystal can be written as follows:

4 $$ {\overline{\chi}}_{k,\lambda . k\hbox{'},\lambda \hbox{'}}==\sqrt{N_0{N}_l}{\displaystyle \sum_s{\overline{\chi}}_{k,\lambda, k\hbox{'},\lambda \hbox{'}}\left({Q}_n, s\right)}{\phi}_{Q_l,{Q}_n, s} $$

Where *N*_0_ and *N*_*l*_ are numbers of unit cells in layer and number of layers in crystal, respectively; besides, in the following, we will write the operator of normal coordinate and tensor of scattering in more simple forms, ${\phi}_{Q_l,{Q}_n, s}={\phi}_{Q, s}$ and ${\overline{\chi}}_{k,\lambda, k\hbox{'},\lambda \hbox{'}}\left({Q}_n, s\right)={\overline{\chi}}_s$.

It was noted in [15, 16] that the intensity of RS is expressed by Fourier component of Green function from tensor of susceptibility of a crystal. In our case, the intensity of light scattering by one unit cell is described by the following expression:

5 $$ {I}_{p\hbox{'},\lambda \hbox{'}, p,\lambda}\sim -\frac{1}{\pi}\left[1+ n\left(\omega \right)\right]\mathrm{I}\mathrm{m}\left\{<<{\chi}_{p\hbox{'},\lambda \hbox{'}, p,\lambda}(t);{\chi}_{p\hbox{'},\lambda \hbox{'}, p,\lambda}^{+}(0)>{>}_{\omega}\right\}=-\frac{1}{\pi}\left[1+ n\left(\omega \right)\right]\mathrm{I}\mathrm{m}\left\{{\displaystyle \sum_{s, s\hbox{'}}{\overline{\chi}}_s{\overline{\chi}}_{s\hbox{'}}^{\ast }}<<{\phi}_{Q, s}(t);{\phi}_{Q, s\hbox{'}}^{+}(0)>{>}_{\omega}\right\}. $$

It is seen from the last line of Eq. (5) that intensity is expressed by the Fourier component of the retarded Green functions from operators of the normal coordinate (for convenience all indexes besides of ones describing the phonon states of layer were omitted and wave vector $\overrightarrow{Q}={\overrightarrow{Q}}_l+{\overrightarrow{Q}}_n\to 0$ is supposed to be small).

In following consideration, we will write:

6 $$ {G}_{s, s\hbox{'}}\left( Q,\omega \right)=<<{\phi}_{Q, s}(t);{\phi}_{Q, s\hbox{'}}^{+}(0)>{>}_{\omega} $$

### Hamiltonian and Equations for Green Functions

Potential energy of crystal vibrations, *V*(*l*, *n*), can be written as series on atom deviations from an equilibrium position, ${u}_{l, n,\alpha}^k$; therefore, the crystal energy in harmonic approximation is written as follows:

7 $$ E={\displaystyle \sum_{l, n, k,\alpha}\frac{{\left({\overset{.}{u}}_{l, n,\alpha}^k\right)}^2}{2{m}_k}}+\frac{1}{2}{\displaystyle \sum_{\begin{array}{l} l, n, k,\alpha, \\ {}\kern1em  n\hbox{'}, k\hbox{'},\beta \end{array}} V"\left({}_{l, n, k,\alpha}^{k\hbox{'}, n\hbox{'},\beta}\right){u}_{l, n,\alpha}^k{u}_{l, n\hbox{'},\beta}^{k\hbox{'}}\Big)}+\frac{1}{2}{\displaystyle \sum_{\begin{array}{l} l, l\hbox{'}\ne l, n, k,\alpha, \\ {}\kern2em  n\hbox{'}, k\hbox{'},\beta \end{array}} V"\left({}_{l, n, k,\alpha}^{l\hbox{'}, k\hbox{'}, n\hbox{'},\beta}\right){u}_{l, n,\alpha}^k{u}_{l\hbox{'}, n\hbox{'},\beta}^{k\hbox{'}}\Big)} $$

In a crystal with identical layers, the phonon frequencies are independent on index layer *l* but depend only on quantum states: ${\omega}_{q,{s}_j}^l\to {\omega}_{q, s}$, *s*_*l*_ → *s*. Besides, interlayer interaction is a function of space between layers, $\tilde{V}\left({}_{s_l,{s}_{l"}, k}^{l, l"}\right)\to \tilde{V}\left({}_{s, s\hbox{'}, k}^{l- l"}\right)$. Therefore, after Fourier transformation, the Eq. (7) can be written as follows (more details see in Additional file 1):

8 $$ H={\displaystyle \sum_{q, p, s}{\omega}_{q, s}}{b}_{q, p, s}^{+}{b}_{q, p, s}+\frac{1}{2}{\displaystyle \sum_{q, p, s, s\hbox{'}}\tilde{V}"\left({}_{s, s\hbox{'}, q}^p\right){\phi}_{q, p, s}{\phi}_{q, p, s\hbox{'}}^{+}} $$

In value $\tilde{V}"\left({}_{s, s\hbox{'}, q}^p\right)$, the down indexes (*s*, *s*′, *q*) characterize the quantum states of layer, but upper one (*p*) points out on the transmission of excitation between layers due to their interaction.

Operators of normal coordinate *ϕ*_*q*,*p*,*s*_ and momentum *π*_*q*,*p*,*s*_ are expressed by creation-annihilation phonons operators of layer, ${b}_{q, p, s}^{+},\kern0.5em {b}_{q, p, s}$, by relations:

9 $$ {\phi}_{q, p, s}=\frac{1}{\sqrt{2}}\left({b}_{q, p, s}+{b}_{- q,- p, s}^{+}\right),\kern0.5em {\pi}_{q, p, s}=\frac{1}{\sqrt{2}}\left({b}_{q, p, s}^{+}-{b}_{- q,- p, s}\right) $$

Fourier components of GF in layer type crystal were defined by Eq.(6), but it is more convenient to consider the GF of more general form:

10 $$ {G}_{k, p, s, k\hbox{'}, p\hbox{'}, s\hbox{'}}(t)=<<{\phi}_{k, p, s}(t);{\phi}_{k\hbox{'}, p\hbox{'}, s\hbox{'}}^{+}(0)>> $$

The equation for such GF looks as follows:

11 $$ i\frac{\partial }{\partial t}{G}_{k, p, s, k\hbox{'}, p\hbox{'}, s\hbox{'}}(t)=\delta (t)<\left[{\phi}_{k, p,, s}(0);{\phi}_{k\hbox{'}, p\hbox{'},, s\hbox{'}}^{+}(0)\right]>+<< i\frac{\partial }{\partial t}{\phi}_{k, p,, s}(t);{\phi}_{k\hbox{'}, p\hbox{'}, s\hbox{'}}^{+}(0)>> $$

Because commutators [*ϕ*_*k*,*p*,*s*_(0); *ϕ*_*k* ',*p* ',*s* '_(0)] = 0 and [*ϕ*_*k*,*p*,*s*_; *H*_int_] = 0 equation for GF is described by simple relation:

12 $$ i\frac{\partial }{\partial t}{G}_{k, p, s, k\hbox{'}, p\hbox{'}, s\hbox{'}}(t)=-{\omega}_{k, s}<<{\pi}_{- k,- p,, s}(t);{\phi}_{k\hbox{'}, p\hbox{'}, s\hbox{'}}^{+}(0)>> $$

The equation for new GF arising in Eq. (12) is likely obtained and looks as follows:

13 $$ i\frac{\partial }{\partial t}<<{\pi}_{- k,- p, s}(t);{\phi}_{k\hbox{'}, p\hbox{'}, s\hbox{'}}^{+}(0)>>==-\delta (t){\delta}_{k, k\hbox{'}}{\delta}_{p, p\hbox{'}}{\delta}_{s, s\hbox{'}}-{\omega}_{k, s}<<{\phi}_{k, p, s}(t);{\phi}_{k\hbox{'}, p\hbox{'}, s\hbox{'}}^{+}(0)>>-{\displaystyle \sum_{s"}\tilde{V}\left({}_{s, s", k}^p\right)}<<{\phi}_{k, p, s"}(t);{\phi}_{k\hbox{'}, p\hbox{'}, s\hbox{'}}^{+}(0)>> $$

After Fourier transformation of Eqs. (12, 13), we obtain the system of coupled equations for Fourier component of GF which can be reduced to one simple equation

14 $$ {\displaystyle \sum_{s"}\left\{\left({\omega}^2-{\omega}_{k, s"}^2\right){\delta}_{s, s"}-{\omega}_{k, s}\tilde{V}\left({}_{s, s", k}^p\right)\right\}}{G}_{k, p, s", s\hbox{'}}\left(\omega \right)={\omega}_{k, s}{\delta}_{s, s\hbox{'}} $$

In Eq. (14), we took into account that nonzero solution is possible if conservation law for wave vectors *k* = *k* ', *p* = *p* ' takes place.

### Spectral Dependence of Raman Scattering

If only two states (*s*_0_, *s*_1_) of layers interact one with another, Eq. (14) results in the following solution of equation (in the following consideration we will mean that indexes describing the components of wave vector of crystal excitations *k*, *p* → 0; because, Eq. (5), both wave vectors are connected with corresponding components of incident radiation $\overrightarrow{Q}={\overrightarrow{Q}}_l+{\overrightarrow{Q}}_n\to 0$)

15 $$ {G}_{s_0{s}_0}=\frac{\omega_{s_0}{\varDelta}_{s_1{s}_1}}{\varDelta},{G}_{s_1{s}_0}=\frac{-{\omega}_{s_0}{D}_{s_1{s}_0}}{\varDelta} $$

16 $$ {G}_{s_1{s}_1}=\frac{\omega_{s_1}{\varDelta}_{s_0{s}_0}}{\varDelta},{G}_{s_0{s}_1}=\frac{-{\omega}_{s_1}{D}_{s_0{s}_1}}{\varDelta} $$

Where we used the designations

17 $$ \varDelta ={\varDelta}_{s_0{s}_0}{\varDelta}_{s_1{s}_1}-{D}_{s_1{s}_0}{D}_{s_0{s}_1} $$

18 $$ {\varDelta}_{s s\hbox{'}}=\left({\omega}^2-{\omega}_{k, s}^2\right){\delta}_{s, s\hbox{'}}+\left[-{\omega}_{k, s}\tilde{V}\left({}_{s, s\hbox{'}, k}^p\right)\right], $$

19 $$ {D}_{s s\hbox{'}}=\left[-{\omega}_{k, s}\tilde{V}\left({}_{s, s\hbox{'}, k}^p\right)\right] $$

Insertion of Eqs. (15, 16) and Eq. (17) into Eq. (5) results in the following relation:

20 $$ {I}_{p\hbox{'},\lambda \hbox{'}, p,\lambda}\sim -\frac{1}{\pi}\left[1+ n\left(\omega \right)\right]\mathrm{I}\mathrm{m}\left\{\frac{1}{\varDelta}\left[{\overline{\chi}}_{s_0}{\overline{\chi}}_{s_0}^{\ast}\left({\omega}_{s_0}{\varDelta}_{s_1{s}_1}\right)+{\overline{\chi}}_{s_0}{\overline{\chi}}_{s_1}^{\ast}\left(-{\omega}_{s_1}{D}_{s_0{s}_1}\right)++{\overline{\chi}}_{s_1}{\overline{\chi}}_{s_0}^{\ast}\left(-{\omega}_{s_0}{D}_{s_1{s}_0}\right)+{\overline{\chi}}_{s_1}{\overline{\chi}}_{s_1}^{\ast}\left({\omega}_{s_1}{\varDelta}_{s_0{s}_0}\right)\right]\right\}. $$

For simplicity, we will assume that the RS tensor components are real, ${\overline{\chi}}_s={\overline{\chi}}_s^{\ast }$. Now, in order to take into account the damping of phonon excitations, we will consider the frequency as a complex value, *ω* → *ω* + *iγ*; therefore, all values depending on frequency in numerator and denominator became complex ones, in particular:

21 $$ {\varDelta}_{s_0{s}_0}=\left({\omega}^2-{\gamma}^2-{\omega}_{s_0}^2+{D}_{s_0{s}_0}\right)+ i2\omega \gamma ={\tilde{\varDelta}}_{s_0{s}_0}+ i2\omega \gamma, $$

22 $$ {\varDelta}_{s_1{s}_1}=\left({\omega}^2-{\gamma}^2-{\omega}_{s_1}^2+{D}_{s_1{s}_1}\right)+ i2\omega \gamma ={\tilde{\varDelta}}_{s_1{s}_1}+ i2\omega \gamma $$

After separating the imaginary part in Eq. (20), the intensity of RS in final form can be written as follows:

23 $$ {I}_{p\hbox{'},\lambda \hbox{'}, p,\lambda}\sim \frac{1}{\pi}\left[1+ n\left(\omega \right)\right]\times \frac{2\omega \gamma \left\{{\omega}_{s_0}\left[{\left({\overline{\chi}}_{s_0}{\tilde{\varDelta}}_{s_1{s}_1}-{\overline{\chi}}_{s_1}{D}_{s_0{s}_1}\right)}^2+4{\overline{\chi}}_{s_0}^2{\omega}^2{\gamma}^2\right]+{\omega}_{s_1}\left[{\left({\overline{\chi}}_{s_1}{\tilde{\varDelta}}_{s_0{s}_0}-{\overline{\chi}}_{s_0}{D}_{s_0{s}_1}\right)}^2+4{\overline{\chi}}_{s_1}^2{\omega}^2{\gamma}^2\right]\right\}}{{\left[{\tilde{\varDelta}}_{s_0{s}_0}{\tilde{\varDelta}}_{s_1{s}_1}-4{\omega}^2{\gamma}^2-{D}_{s_1{s}_0}{D}_{s_0{s}_1}\right]}^2+{\left[2\omega \gamma \left({\tilde{\varDelta}}_{s_0{s}_0}+{\tilde{\varDelta}}_{s_1{s}_1}\right)\right]}^2}. $$

Eq. (23) and Eqs. (21, 22), (18, 19) show that RS intensity has an enough complicated dependence on the frequency *ω*, and on interaction between layers described by value $\tilde{V}\left({}_{s, s\hbox{'}, k}^p\right)$, Eqs. (18, 19).

Resonance frequencies are defined from the first term in denominator of Eq. (23) (if *γ* → 0) and according to Eqs. (21, 22) are equal:

24 $$ {\omega}^2-{\gamma}^2=\frac{1}{2}\left\{\left[\left({\omega}_{s_0}^2-{D}_{s_0{s}_0}\right)+\left({\omega}_{s_1}^2-{D}_{s_1{s}_1}\right)\right]\pm \sqrt{{\left[\left({\omega}_{s_0}^2-{D}_{s_0{s}_0}\right)-\left({\omega}_{s_1}^2-{D}_{s_1{s}_1}\right)\right]}^2+16{\omega}^2{\gamma}^2+4{D}_{s_0{s}_1}{D}_{s_1{s}_0}}\right\} $$

It is seen from Eq. (24) and Eq. (18, 19), if interlayer interaction $\tilde{V}\left({}_{s_0,{s}_1, k}^p\right)=0,\kern0.5em \left(\gamma \to 0\right)$, that the frequencies are equal: ${\omega}_{+}={\omega}_{s_0}$ and ${\omega}_{-}={\omega}_{s_1}$ (if ${\omega}_{s_1}<{\omega}_{s_0}$), respectively. By inclusion of interlayer interaction, $\tilde{V}\left({}_{s_0,{s}_1, k}^p\right)\ne 0$ the intermixing of layer fundamental vibrations occurs, and frequencies *ω*_+_ and *ω*_−_ are shifted into different sides. It is also seen from Eq. (24) and Eqs. (18, 19) that intra-layer frequencies ${\omega}_{s_0},\kern0.5em {\omega}_{s_1}$ can increase, if interlayer interaction between states $\tilde{V}\left({}_{s_0,{s}_1, k}^p\right)>0$.

## Results and Discussion

The estimation of interlayer interaction parameters can be made on the base of results of phonon theoretical calculations for one layer and bulk *MoS*_2_ crystal obtained in work [17]. The actual phonon frequencies for one-layer *MoS*_2_, of *D*_3*h*_ point group symmetry, are the following: ${\omega}_{A_1^{\hbox{'}}}$ = 410.3 cm^−1^ and ${\omega}_{E_1^{\hbox{'}}}$ = 391.7 cm^−1^; however, the corresponding ones for the bulk *MoS*_2_ crystal with *D*_6*h*_ point group have the following values ${\omega}_{A_{1 g}}$ = 412 cm^−1^ and ${\omega}_{E_{2 g}}$ = 387.8 cm^−1^ what is very close to former phonon pair. It means that the change of frequencies in bulk due to the interlayer interaction is near 3–4 cm^−1^ (the precision of numerical calculations is near ~1.5 cm^−1^ what follows from calculated difference ${\omega}_{E_{1 g}}-{\omega}_{E_{1 u}}=1.6$ cm^−1^, [17], Table 2).

Experiments however showed [4] that observed frequencies ${\omega}_{A_1^{\hbox{'}}}\approx$403 cm^−1^ and ${\omega}_{E_1^{\hbox{'}}}\approx$384 cm^−1^ for one layer differ significantly from ones predicted by theory (${\omega}_{A_1^{\hbox{'}}, calc}$ − ${\omega}_{A_1^{\hbox{'}}, \exp }=7.3$ cm^−1^). One of the reasons of such difference can be connected with anharmonic interactions, which are in monolayer and which were not taken into account in theoretical calculations. Indeed, from work [17], (Fig. 2, upper panel) follows that combination tones *ω*_*LA*_(*q*_*M*_) + *ω*_*LA*_(−*q*_*M*_) ≈ *ω*_*LA*_(*q*_*K*_) + *ω*_*LA*_(−*q*_*K*_) ≈470 cm^−1^ are fully symmetric and have frequency greater than ${\omega}_{A_1^{\hbox{'}}}$ (for points *K*, *M* symmetry group can be subgroup of *D*_3*h*_, for example, *C*_3*h*_). Therefore, both vibrations can take part in Fermi resonance (FR) interaction, and as a result, the calculated fundamental band, ${\omega}_{A_1^{\hbox{'}}}$ =410.3 cm^−1^, should be slightly shifted down to experimentally observed meaning ${\omega}_{A_1^{\hbox{'}}}\approx$403 cm^−1^. Besides, a weak band of combination tone (CT) appears at ${\omega}_{CT}^{\hbox{'}}\approx$473 cm^−1^ is being slightly shifted to high frequency side according to FR rules. This pair of monolayer phonons symmetry ${A}_1^{\hbox{'}}$ will give rise to additional intermixing of them in crystal at overjumping ones to other layers at growth of their numbers. Similar shift should occur for monolayer fundamental vibration ${\omega}_{E_1^{\hbox{'}}}$ = 391.7 cm^−1^ due to FR with CT *ω*_*LA*_(*q*_*K*_) + *ω*_*TA*_(−*q*_*K*_) ≈420 cm^−1^ having symmetry, ${E}_1^{"}\otimes A"={E}_1^{\hbox{'}}$, for group *D*_3*h*_. The calculated band ${\omega}_{E_1^{\hbox{'}}}$ = 391.7 cm^−1^ is also shifted to position experimentally observed at ${\omega}_{E_1^{\hbox{'}}}\approx$383 cm^−1^, [4] due to noted FR. New doublet of bands of ${E}_1^{\hbox{'}}$ symmetry will also give rise to additional intermixing of both phonons at growth of layer numbers. Experiment shows, however, that if the number of layers is >4 the position and intensities of discussed bands of thin crystal become like that ones observed for bulk *MoS*_2_ [4, 5]. Thus, the discussed above bands (${A}_1^{\hbox{'}}$, ${E}_1^{\hbox{'}}$) of monolayer with growing up of the layers are transformed into new pair with symmetries, *A*_1*g*_ and ${E}_{2 g}^1$, characteristic for bulk crystal. It is clear that on going from monolayer to bulk the properties of bulk *MoS*_2_ are particularly added to parameter $\tilde{V}\left({}_{s_0,{s}_1, k}^p\right)$, by anharmonicity of bulk, because symmetries of monolayer and bulk *MoS*_2_ are different.

**Fig. 1:**
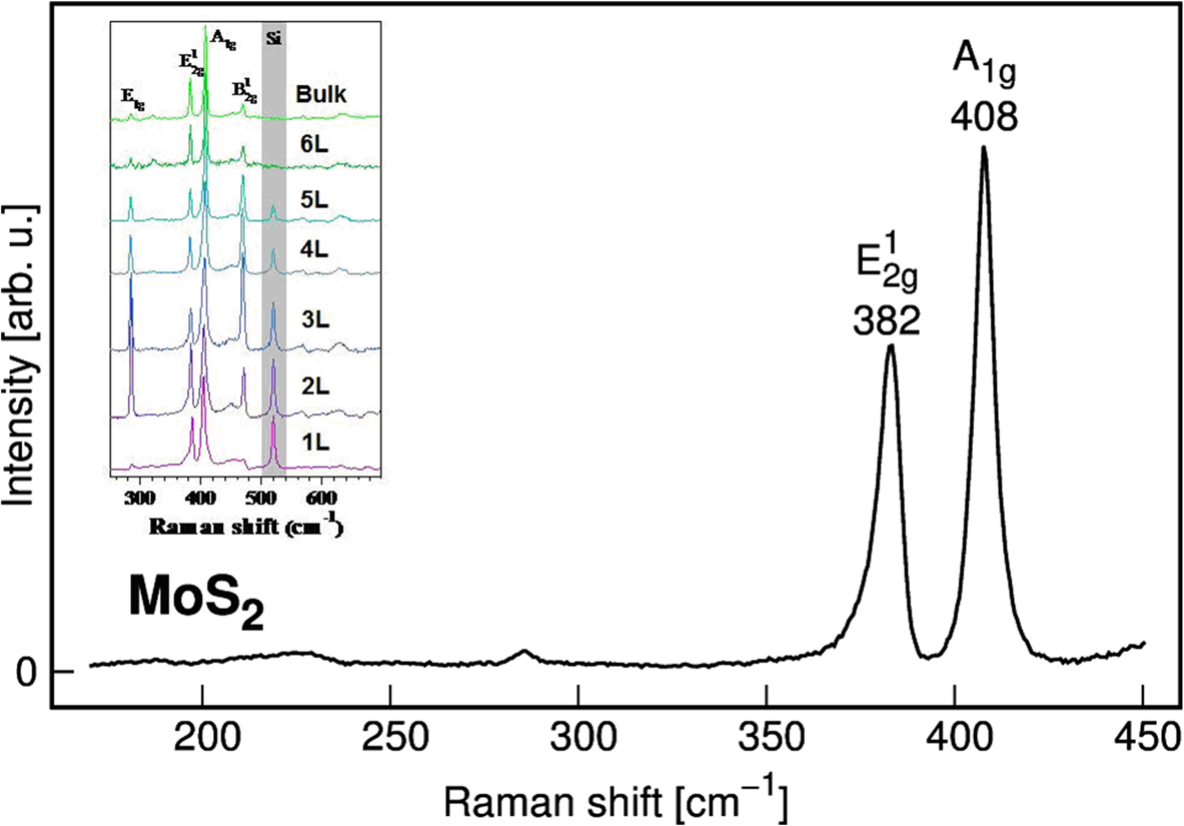
Raman spectra of bulk and nanothin *MoS*_2_. Change of intensities as function of number of layers is shown in insertion

In the case of bulk *MoS*_2_ discussed above, the *A*_1*g*_ and ${E}_{2 g}^1$ bands are observed as two strong fundamental ones, Fig. 1. Insertion in Fig. 1, taken from our work [5], shows the transformation of these band intensities as function of layer numbers (thickness). It was shown in [5] that for atomically thin crystal *MoS*_2_ consisting of the few layers that there are some different forbidden rules for even and odd layer numbers. That is clearly seen for ratio of intensities for cases 1L, 3L and 2L in insertion. In the last case, the intensity of ${E}_{2 g}^1$ band increases significantly. For other cases beginning from thickness 4L and up to bulk, the ratio of band intensities ${E}_{2 g}^1$ and *A*_1*g*_ is practically unchanged. The transformation of other bands, *E*_1*g*_ and ${B}_{2 g}^1$, allowed for atomically thin crystals but disappearance for bulk case is also seen in insertion.

One can also note in Fig. 1 that experimentally observed frequencies for bulk *MoS*_2_ are markedly different from calculated ones, *ω*(*A*_1*g*_) = 412 cm^−1^ and $\omega \left({E}_{2 g}^1\right)$ = 388 cm^−1^. Bands *ω*(*A*_1*g*_) and $\omega \left({E}_{2 g}^1\right)$ have different symmetry and so direct interaction of corresponding vibrations by anharmonicity what result in the change of their frequencies is impossible. However, there is the reason giving rise to difference between calculation and experiment: it is the existence in *MoS*_2_ bulk crystal, the CT, $\omega \left({E}_{2 g}^1\right)\pm \omega \left({E}_{2 g}^2\right)$ = (388 ± 35) cm^-1^ close placed to discussed fundamental frequencies, ([17] Table 1). These CT can interact with fundamental vibrations *A*_1*g*_ and *E*_2*g*_ symmetry due to FR, admitted for *D*_6*h*_ point group symmetry of bulk *MoS*_2_ by the relation *E*_2*g*_ × *E*_2*g*_ = *A*_1*g*_ + *E*_2*g*_. As a result, both fundamental ones should be shifted.

**Fig. 2:**
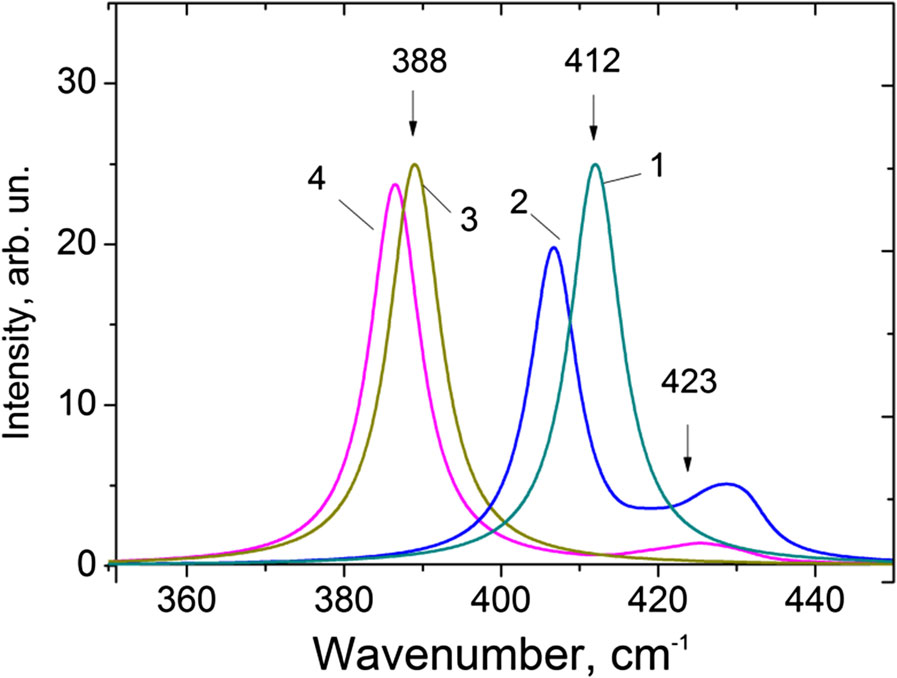
Shift of calculated fundamental bands for bulk *MoS*_2_. Parameters obtained at FR: ${\omega}_{A_{1 g, calc}}$ = 412 cm^−1^ and ${\omega}_{E_{2 g, calc}}$ = 388 cm^−1^, to experimental meanings, ${\omega}_{A_{1 g, \exp }}$ = 408 cm^−1^ (*curve 2*) and ${\omega}_{E_{2 g, \exp }}$ = 382 cm^−1^ (*curve 4*) due to FR; *Γ*_+_ = *Γf*(*T*), $f\left( T=300 K\right)=\left[1+ n\left({E}_{2 g}^2\right)+ n\left({E}_{2 g}^1\right)\right]=3.8$; two studied FR correspond to different anharmonic constants: *Γ*(*A*_1*g*_, *CT*) = 2.4 cm^−1^, $\varGamma \left({E}_{2 g}^1, CT\right)=4.5$ cm^−1^; (CT in Fig. 2 is described by enough broad band due to the dispersion of phonons [18, 19] and the centre of gravity CT is shown by *arrow* at *ω* = 423 cm^−1^; because only shift of bands due to FR is important for both fundamental bands, the same initial intensity is taken)

Position and intensities of *A*_1*g*_ and *E*_2*g*_ bands depend on anharmonic constant *Γ* responsible for FR interaction [18, 19]. Effect of FR on fundamentals is shown in Fig. 2. As a result of anharmonic interaction of the fundamental *ω*(*A*_1*g*_) = 412 cm^−1^ with CT $\omega \left({E}_{2 g}^1\right)$ + $\omega \left({E}_{2 g}^2\right)$ = 423 cm^−1^, the first is shifted down to meaning *ω*(*A*_1*g*_) ≈408 cm^−1^, Fig. 2, curves 1 and 2. Due to quite great space between fundamental *ω*(*A*_1*g*_) and combination tones $\omega \left({E}_{2 g}^1\right)-\omega \left({E}_{2 g}^2\right)$ = 353 cm^−1^, the influence of this CT on fundamental can be neglected.

The other fundamental band $\omega \left({E}_{2 g}^1\right)$ = 388 cm^−1^ can also interact with these combination tones $\omega \left({E}_{2 g}^1\right)\pm \omega \left({E}_{2 g}^2\right)$ = (388 ± 35) cm^−1^, but such an interaction is more complicated, because two CT are placed around this fundamental band at the same distances: $\omega \left({E}_{2 g}^1\right)+\omega \left({E}_{2 g}^2\right)$ = 423 cm^−1^ and $\omega \left({E}_{2 g}^1\right)-\omega \left({E}_{2 g}^2\right)$ =353 cm^−1^. According to the theory of FR in crystals [18, 19], intensity of Raman scattering (absorption) and shift of interacting bands are described by renormalized constant *Γ*. For each of the studied cases, this constant is given by the following relations: for the first case of FR, $\varGamma \to {\varGamma}_{+}=\varGamma \left[1+ n\left({E}_{2 g}^2\right)+ n\left({E}_{2 g}^1\right)\right]$ and for the second one, $\varGamma \to {\varGamma}_{-}=\varGamma \left[ n\left({E}_{2 g}^2\right)- n\left({E}_{2 g}^1\right)\right]$, where $n\left({E}_{2 g}^2\right),\kern0.5em  n\left({E}_{2 g}^1\right)$ are occupation numbers of the corresponding phonons. Because *Γ*_+_ > *Γ*_−_, the fundamental band of *MoS*_2_, $\omega \left({E}_{2 g}^1\right)$ = 388 cm^−1^ due to more strong FR interaction with CT $\omega \left({E}_{2 g}^1\right)+\omega \left({E}_{2 g}^2\right)$, located upper fundamental $\omega \left({E}_{2 g}^1\right)$, is shifted slightly below to experimentally observed meaning, $\omega \left({E}_{2 g}^1\right)\approx$382 cm^−1^, Fig. 2, curves 3 and 4. Thus, taking into consideration the anharmonic interactions, the better correlation of calculation and experiment can be obtained.

One can note that the frequencies $\omega \left({E}_{2 g}^1\right)$ and *ω*(*A*_1*g*_) are caused by presence of two layers in the crystal unit cell; therefore, anharmonic interactions in bulk *MoS*_2_ are also related with these two layers. However, according to Fig. 1 of present work and Figs. 4–6 work of [5], the number of layers has influence on the spectrum. For example, the odd or even number of layers in nanothin crystals is very important for intensities of bands, but it is not so important for their frequencies which are changing smoothly enough. Therefore, for description of frequencies dependence of the discussed $\omega \left({E}_{2 g}^1\right)$ and *ω*(*A*_1*g*_) bands on the layer numbers, one can use the monolayer properties of *MoS*_2_.

**Fig. 3:**
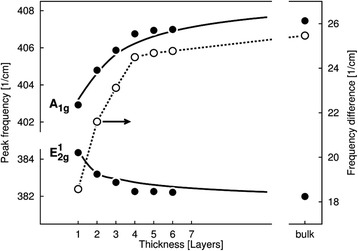
The change of intra-layer phonon frequencies ${\omega}_{A_1^{\hbox{'}}}\approx$403 cm^−1^ and ${\omega}_{E_1^{\hbox{'}}}\approx$384 cm^−1^ with increasing of layer numbers. Studied monolayer phonon frequencies are transformed into bulk ones *ω*(*A*_1*g*_) ≈408 cm^−1^ and $\omega \left({E}_{2 g}^1\right)\approx$382 cm^−1^ correspondingly; *thick dots* are experimental dependences and *circles* corresponds to difference between frequencies *ω*(*A*_1*g*_) and $\omega \left({E}_{2 g}^1\right)$ for each layer; *solid curves* describe the theoretical dependences; fitting parameters (in cm^−1^) for *ω*(*A*_1*g*_), ${V}^{s_0,{s}_0}$ = 8, ${V}^{s_0,{s}_1}$ = ${V}^{s_1,{s}_0}$ = 6, ${V}^{s_1,{s}_1}$ = 3; for $\omega \left({E}_{2 g}^1\right)$, ${V}^{s_0,{s}_0}$ = 5, ${V}^{s_0,{s}_1}$ = ${V}^{s_1,{s}_0}$ = 4, ${V}^{s_1,{s}_1}$ = 2; (experimental data are taken from [4])

According to [4], the experimental values of monolayer frequencies are equal ${\omega}_{A_1^{\hbox{'}}}\approx$403 cm^−1^ and ${\omega}_{E_1^{\hbox{'}}}\approx$384 cm^−1^ as what is shown in Fig. 3 by thick points for *L* = 1. Then, on going from monolayer to bulk *MoS*_2_, two noted bands are transformed into other doublet ${\omega}_{A_1^{\hbox{'}}}\to \omega \left({A}_{1 g}\right)\approx$408 cm^−1^ and ${\omega}_{E_1^{\hbox{'}}}\to \omega \left({E}_{2 g}^1\right)\approx$382 cm^−1^. (These limit frequencies can be slightly changed on 1–2 cm^−1^ at different experiments [5]). It is also seen from Fig. 3 that frequencies for monolayer ${\omega}_{A_1^{\hbox{'}}}$ and ${\omega}_{E_1^{\hbox{'}}}$ are changed differently from transition to bulk *MoS*_2_: ${\omega}_{A_1^{\hbox{'}}}$ increases but ${\omega}_{E_1^{\hbox{'}}}$ decreases. Such diminish of ${\omega}_{E_1^{\hbox{'}}}$ with growth of layer numbers was explained in [17] as “anomalous Davydov splitting”, and it was related with effect of dielectric tensor [7]. In the present work, we show that different behaviour of bands can be connected with two effects: with increasing of interlayer interaction growing up of layer numbers and also with appearing of additional weak bands (CT) at frequencies ~${\omega}_{CT}\left({E}_1^{\hbox{'}}\right)\approx$425 cm^−1^ and ${\omega}_{CT}\left({A}_1^{\hbox{'}}\right)\approx$475 cm^−1^, what is caused by FR discussed above for monolayer *MoS*_2_. Increasing of interaction between these combination tones of corresponding fundamentals ${\omega}_{E_1^{\hbox{'}}}$ and ${\omega}_{A_1^{\hbox{'}}}$ with growing up of layer numbers can be real reason of “anomalous Davydov splitting” origin. Theoretical dependences presented in Fig. 3 shows that change of frequencies depends on the values $\tilde{V}\left({}_{s_0,{s}_1, k}^p\right)$ connected with concrete quantum states *s*_0_ and *s*_1_. Because one of these states, *s*_0_, in our description is connected with fundamental vibration and other, *s*_1_, characterizing the CT, we obtain some special form of FD resonance for layer type crystal.

It should be noted that at comparison of theoretical calculations with experiment, [17] Fig. 6, the greatest difference between both results was observed at *L* = 1 for both ${\omega}_{A_1^{\hbox{'}}}$ and ${\omega}_{E_1^{\hbox{'}}}$ vibrations. Because in this case, the interlayer interaction is absent, $\tilde{V}\left({}_{s, s\hbox{'},, k}^p\right)$ = 0, the reason of such difference can be related with effect of anharmonic interaction and FR in the layer what result in the shift down of calculated frequencies ${\omega}_{A_1^{\hbox{'}}}$ = 410.3 cm^−1^ and ${\omega}_{E_1^{\hbox{'}}}$ = 391.7 cm^−1^. For band ${\omega}_{A_1^{\hbox{'}}}$, the marked difference between theory and experiments is observed also for *L* = 2 and 3. It is clear that with growth of the layer numbers the value of interlayer interaction increases and the intralayer anharmonic interaction effect, which was not taken into account in theory [17], became not so important. Therefore, the agreement of theoretical calculations with experiment for big L is better. The effects of influence of the intramolecular anharmonism on Davydov splitting were studied before in works [11, 12, 14] for molecular type crystals.

Increasing of parameters $\tilde{V}\left({}_{s, s\hbox{'}, k}^p\right)\to {V}^{s, s\hbox{'}},\kern0.62em \left( k, p\to 0\right)$ describing the interlayer interaction with growth of layer numbers can be seen from the following expression (more details are presented in Additional file 2)

25 $$ {\tilde{V}}_{cryst}^{s, s\hbox{'}}={V}^{s, s\hbox{'}}{\displaystyle \sum_{n=1}^N\frac{1}{n^{1+\alpha}}},\alpha >0 $$

Fitting of the dependence described by Eq. (24) with using Eq. (25) to experiment [4] gives the possibility to obtain the parameters of interlayer interactions *V* ^*s*,*s* '^. For *MoS*_2_ crystal, the best fit is observed at *α* = 0.45.

One can note that change of layer frequencies at transition from monolayer to bulk was recently studied in layer type crystal V_2_O_5_. The situation with this crystal is more complicated [20] because it has enough low crystal symmetry, point group D_2h_, 39 optical vibrations and one dimensional representation. But for this crystal, there are some features related with product of irreduced representations, for example, *B*_1*g*_ ⊗ *B*_1*g*_ =, *B*_1*u*_ ⊗ *B*_1*u*_ = and *B*_3*u*_ ⊗ *B*_3*u*_ = *A*_1*g*_. Therefore, many combination tones appear which can interact by anharmonicity with fundamental vibrations of corresponding symmetry and which result in shifting them to different sides. That depends on their initial frequencies: *ω*_*fund*_ > *ω*_*comb. tone*_ or contrary. In particular, it is seen in Fig. 9 work [20]. The highest band of monolayer, *B*_3*u*_ symmetry, is located at 1088 cm^−1^, but at some upper of this fundamental, the combination tone *ω*(*B*_3*u*_) + *ω*(*A*_1*g*_) = (747 + 471) cm^−1^ of the same symmetry *B*_3*u*_ is placed. As a result of going from monolayer to bulk, the interlayer and anharmonic interactions increase and the fundamental band is shifted down. But if two CT interacting with fundamental are located upper and lower of it, the last can be immobile at transition from layer to bulk. Such type of FR was recently discussed in work [21].

## Conclusions

In the present article on the example of MoS_2_ layer type crystal, we show that change of phonon bands position, in-plane ${E}_{2 g}^1$ and out-of-plane *A*_1*g*_, as a function of number of layers for thin layer crystals can be understood if one uses the Hamiltonian written in the form separating the strong intralayer and weak interlayer interactions. It is also shown that taking into account anharmonic effects of layer together with wdW interlayer interaction gives possibility to describe correctly enough the observed changes of phonon frequencies with increasing of layer numbers. Estimation of parameters describing the interlayer interaction was made by comparison of theory with experiment, and it was obtained that these values for MoS_2_ layer crystal are significantly smaller than studied intralayer frequencies.

## Additional files

Additional file 1: Hamiltonian of layer-type crystal. (DOCX 180 kb)
Additional file 2: Van der Waals interaction between layers. (DOCX 102 kb)

## Abbreviations

CT: Combination tone
DS: Davydov splitting
FD: Fermi-Davydov
FR: Fermi resonance
GF: Green function
RS: Raman scattering
vdW: Van der Waals
