# Supplementary material for: Theoretical and Experimental Study of Phonon Spectra of Bulk and Nano-Sized MoS2 Layer Crystals
Source: Nanoscale Res Lett. 2017 Jan 31;12:82. doi: 10.1186/s11671-016-1808-8 (PMC5285300; doi:10.1186/s11671-016-1808-8)
Supplement: Additional file 1: — Hamiltonian of layer-type crystal. (DOCX 180 kb) [file 11671_2016_1808_MOESM1_ESM.docx]

SUPPLEMENTARY MATERIALS to the manuscript “Theoretical and experimental study of phonon spectra of bulk and nano-sized MoS_2_ layer crystals” (by A.M. Yaremko, V.O. Yukhymchuk, Yu. A. Romanyuk, J. Baran, M. Placidi**)**

**Hamiltonian of layer-type crystal**

Potential energy of crystal vibrations, $V(l,n)$, can be written as series on atom deviations from an equilibrium position, $u_{l,n,\alpha}^{k}$. Energy of crystal in harmonic approximation then can be written as follows:

$\frac{1}{2}\sum_{\begin{matrix} l,l'\neq l,n,k,\alpha, \\ n',k',\beta\end{matrix}} V"(_{l,n,k,\alpha}^{l',k',n',\beta})u_{l,n,\alpha}^{k}u_{l',n',\beta}^{k'})$ (1)

Here

$V"(_{l,n,k,\alpha}^{l',n',k',\beta})=(\frac{\partial^{2}V}{\partial u_{l,n,\alpha}^{k}\partial u_{l',n',\beta}^{k'}})_{0}$. (2)

The transformation of first two expressions related with the same layer is traditional for lattice dynamic therefore we consider in some details the last term in Eq. (1).

$H_{\mathrm{int}}=\frac{1}{2}\sum_{\begin{matrix} l,l'\neq l,n,k,\alpha, \\ n',k',\beta\end{matrix}} V"(_{l,n,k,\alpha}^{l',n',k',\beta})u_{l,n,\alpha}^{k}u_{l',n',\beta}^{k'})$ $=\frac{1}{2}\sum_{\begin{matrix} l,l'\neq l,n,k,\alpha, \\ n',k',\beta\end{matrix}} V"(_{l,n,k,\alpha}^{l',n',k',\beta})$ $\{\frac{1}{\sqrt{N_{0}}}\sum_{q,s_{l}} [A_{l,\alpha}^{k}(q,s_{l})\exp(iqn)\phi_{q_{l},s_{l}}^{l}] \}\times$ $\times\{\frac{1}{\sqrt{N_{0}}}\sum_{q',s_{l'}} [A_{l',\beta}^{*k'}(q',s_{l'})\exp(-iq'n')\phi_{q',s_{l'}}^{l'}] \}$. (3)

Energy interaction between different unit cells in crystal is the function of difference space between atoms in these cells, therefore one can write

, (4)

$\exp[i(qn-q'n')]=\exp[in(q-q')+iq'(n-n')]$. (5)

Where exponent in Eq. (1) was also transformed.

Therefore substitution the Eqs. (4, 5) into Eq. (3) results in relation

$H_{\mathrm{int}}$ $=\frac{1}{2}\sum_{\begin{matrix} q,s_{l},q',s_{l'}, \\ k,\alpha,k',\beta,l\neq l' \end{matrix}} \{\frac{1}{N_{0}}\sum_{ n,n'} V"[(l-l')+(n-n');k',\beta;k,\alpha]A_{l,\alpha}^{k}(q,s_{l})A_{l',\beta}^{*k'}(q',s_{l'})\phi_{q,s_{l}}^{l}\phi_{q',s_{l'}}^{+l'}\}\times$ $\times\exp[iq'(n-n')+in(q-q')]$. (6)

It is obvious that summation on $n'$ Eq. (6) with exponent$\exp[iq'(n-n')]$ gives rise the expression which is independent on index $n$ and therefore one can make the following summation on index $n$ what results in to delta function, $\delta_{qq'}$. Therefore the Eq. (6) became more simple.

$H_{\mathrm{int}}$ $=\frac{1}{2}\sum_{q,s_{l},s_{l'},l\neq l'} \{\sum_{ k,\alpha,k',\beta} V"[(l-l');k,\alpha;k',\beta]A_{l,\alpha}^{k}(q,s_{l})A_{l',\beta}^{*k'}(q,s_{l'})\}\phi_{q,s_{l}}^{l}\phi_{q,s_{l'}}^{+l'}$=

. (7)

In Eq. (7) we used the designation

$\tilde{V}"(_{q,s_{l},s_{l'}}^{l,l'(\neq l)})=\sum_{ k,\alpha,k',\beta} V"[(l-l');k,\alpha;k',\beta]A_{l,\alpha}^{k}(q,s_{l})A_{l',\beta}^{*k'}(q,s_{l'})$. (8)

If crystal consist of identical layers the indexes which describe the quantum states, $s_{l}$, should not depends on index $l$ layer, therefore we have to change $s_{l}\to s$ in values $A_{l,\alpha}^{k}(q,s_{l})\to A_{\alpha}^{k}(q,s)$. Then change of function allows make the Fourier transformation Eq. (5) as follows:

. (9)

Thus transformation of $H_{\mathrm{int}}$ from Eq. (3) to Eq. (9) shows that in parameter $\tilde{V}"(_{s,s',q}^{p})$ all atom-atom (ion-ion) interactions between atoms of different layers are included on micro level. Dielectric constant can be included phenomenologically as it was made in work [7].

The transformation of first two terms in Eq. (1) is traditional and reduces them to very simple expression for crystal with identical layers, namely

$H_{0}=\sum_{l,q,s_{l}} \omega_{q,s_{l}}^{l}b_{q,s_{l}}^{+l}b_{q,s_{l}}^{l}=$ $\sum_{q,p,s} \omega_{q,s}b_{q,p,s}^{+}b_{q,p,s}$. (10)

The last term in Eq. (10) is obtained due to Fourier transformations because $\omega_{q,s_{l}}^{l}\to\omega_{q,s}$.
