# Supplementary material for: Theoretical and Experimental Study of Phonon Spectra of Bulk and Nano-Sized MoS2 Layer Crystals
Source: Nanoscale Res Lett. 2017 Jan 31;12:82. doi: 10.1186/s11671-016-1808-8 (PMC5285300; doi:10.1186/s11671-016-1808-8)
Supplement: Additional file 2: — Van der Waals interaction between layers. (DOCX 102 kb) [file 11671_2016_1808_MOESM2_ESM.docx]

SUPPLEMENTARY MATERIALS to the manuscript “Theoretical and experimental study of phonon spectra of bulk and nano-sized MoS_2_ layer crystals” (by A.M. Yaremko, V.O. Yukhymchuk, Yu. A. Romanyuk, J. Baran, M. Placidi**)**

**Van der Vaals interaction between layers**

Potential and electric field in point R of space created by dipole $d=el$ located at $R=0$ are described by following relations and respectively. Energy interaction of dipole located unit cell $m$ of layer $i$ with electric field created in this cell by dipole placed in unit cell $k$ of layer $j$ is following:

. (1)

In quantum mechanic interaction between dipole moment transition which characterized by $f,g$ states is given by matrix element from energy described by Eq. (1), namely:

.

We have to study the interaction of all dipole moment transitions $d_{kj}^{g}$of layer $j$ with ones $d_{mi}^{f}$ placed in layer $i$, by other word to of make summation on all unit cells $k$ of layer, $j$.

The second term in Eq. (1) can be written as follows:

. (2)

For simplicity we will suppose that dipole in $i$ layer is located at cell, $m=0$. Therefore at summation on $k$ for fixed number of layers, $i,j$ for values $R_{0i,kj}$ which are greater then lattice constant, for each angle $\theta_{1}, (\theta_{2})$ there is an angle $\approx\pi-\theta_{1},(\pi-\theta_{2})$ for which $\cos(\pi-\theta_{1})=-\cos\theta_{1}$, (the same occurs for angle$\theta_{2}$). Such terms will exclude each other. Therefore the contribution of second term in Eq. (1) will be significantly smaller then first one at summation on $k$. Estimation of sum from the first term in Eq. (1) can be obtained if one takes into account that all unit cells in crystal are identical and . Then average sum interaction of dipole in ($0,i$) cell with $j$ layer can be calculated as follows:

, (3)

$S_{0}=vN_{0}$ is the square of layer.

It is clear that summation on all cells of $i$ layer will result in the similar relation. Eq. (3) shows that layer-layer interaction diminish enough slowly. Full interaction of layer $l$=0 with all other crystal layers *in this approximation* is described by the following expression:

$V_{cryst}^{fg}=V^{fg}\sum_{n=1}^{N} \frac{1}{n}$, $\left|l\right|=na_{0}, n=1,2,3,...N$. (4)

Here $a_{0}$ is the space between neighbouring layers.

It is known if, $N\to\infty$ the series in Eq.(4) diverge $\sum_{k=1}^{N\to\infty} \frac{1}{k}\approx C+\ln N$, and$V_{cryst}^{fg}\to\infty$. On the other hand the series, $\sum_{k=1}^{N} \frac{1}{n^{p}}$, $p>1$, converge (for example, $\sum_{n=1}^{N} \frac{1}{n^{2}}=\frac{\pi^{2}}{6}$). Good approximation for interaction of layer $l$=0 with other crystal layers can be the following relation which also particularly takes into account the contribution of second term in Eq. (1) omitted at summation in Eq. (3)

$V_{cryst}^{fg}=V^{fg}\sum_{n=1}^{N} \frac{1}{n^{1+\alpha}}$, $p=1+\alpha$, $\alpha>0$. (5)

In our case the best fitting occurs for parameter$\alpha=0.45$, Figure 3 of work.
